# Small Extracellular Vesicles: Functions and Potential Clinical Applications as Cancer Biomarkers

**DOI:** 10.3390/life11101044

**Published:** 2021-10-04

**Authors:** Yi Wang, Ruichen Zhao, Xueqiao Jiao, Longyuan Wu, Yuxuan Wei, Fuxiu Shi, Junpei Zhong, Lixia Xiong

**Affiliations:** 1 Department of Pathophysiology, Medical College, Nanchang University, 461 Bayi Road, Nanchang 330006, China; 401442719031@email.ncu.edu.cn (Y.W.); r.zhao@se17.qmul.ac.uk (R.Z.); 6303416020@email.ncu.edu.cn (X.J.); 6300817039@email.ncu.edu.cn (L.W.); y.wei@se18.qmul.ac.uk (Y.W.); 401428820001@email.ncu.edu.cn (F.S.); 401428820017@email.ncu.edu.cn (J.Z.); 2 Jiangxi Province Key Laboratory of Tumor Pathogenesis and Molecular Pathology, 461 Bayi Road, Nanchang 330006, China

**Keywords:** cancer, sEVs, liquid biopsy, clinical applications

## Abstract

Cancer, as the second leading cause of death worldwide, is a major public health concern that imposes a heavy social and economic burden. Effective approaches for either diagnosis or therapy of most cancers are still lacking. Dynamic monitoring and personalized therapy are the main directions for cancer research. Cancer-derived extracellular vesicles (EVs) are potential disease biomarkers. Cancer EVs, including small EVs (sEVs), contain unique biomolecules (protein, nucleic acid, and lipids) at various stages of carcinogenesis. In this review, we discuss the biogenesis of sEVs, and their functions in cancer, revealing the potential applications of sEVs as cancer biomarkers.

## 1. Introduction

Cancer refers to a neoplasm characterized by abnormal and uncontrolled cell growth and high invasive capability. As the second leading cause of death worldwide [1], cancer is recognized as a serious public health problem that must be urgently addressed. Recently, many researchers have discovered evidence that cancer cell activities are largely dependent on extracellular vesicles (EVs), showing that EVs play a crucial role in the development of cancer, including the processes of angiogenesis, epithelial-to-mesenchymal transition (EMT), invasion, metastasis, immunosuppression, and drug resistance [2]. EVs are secreted to establish a favorable microenvironment for better growth through the transportation of various cargoes [3]. For example, cancer cells can domesticate their target cells by transporting RNA and DNA to influence their gene expression [4]. Cancer cells can also alter cell metabolism by sending specific proteins to cells of normal tissue [3].

Based on biogenetic mechanisms, EVs are divided into two major subtypes: plasma membrane-derived ectosomes (microvesicles/microparticles) and endosome-origin small extracellular vesicles (sEVs), with diameter varying from 30 to 100 nm or from 50 to 1000 nm, respectively. A subset of ectosome are referred to as exosomes [5]. Ectosomes are also known as microvesicles, microparticles, or shedding vesicles [6,7]. Here, we use ‘sEVs’ to refer to EVs less than 200 nm in diameter. The size, and physical and chemical characteristics of EVs are detailed in Table 1 and Table 2.

Due to their presence and stability in most body fluids [10,11], tumor-derived sEVs (TEVs) can be used as biomarkers for cancer detection, because the cargo they carry reflects genetic or signaling changes in the cancer cell of origin [12,13,14]. In addition, cancer cells secrete more sEVs than normal cells, which are released into the tumor microenvironment (TME) and circulation [2,15,16,17,18,19]. Furthermore, the contents of sEVs are potential biomarkers (such as microRNA(miRNAs)) which could improve the specificity of cancer diagnosis and prognosis [18,20,21,22,23], and help to manage and predict treatment responses. Therefore, further research into TEVs is needed. In this review, we summarize the processes involved in sEV biogenesis, the roles of sEVs in cancer functions, and their potential clinical applications as predictive biomarkers in various cancers.

## 2. Biogenesis of sEVs

**Figure 1:**
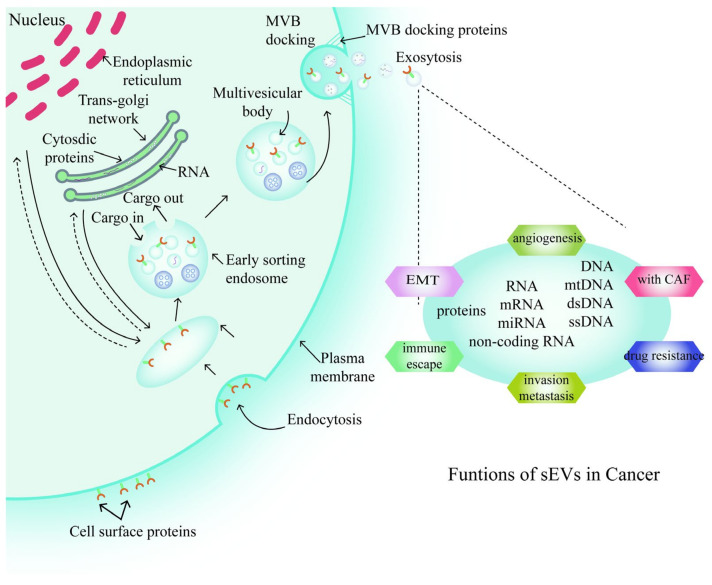
Steps in the formation of small extracellular vesicles (sEVs). Abbreviations: MVB, multivesicular body; EMT, epithelial-to-mesenchymal transition; CAF, cancer-associated fibroblast.

Exosomes are a type of nanoscale, endogenously derived vesicle with phospholipid bilayers, and widely known as mediators of cell-to-cell communication by transferring cargo from one cell to another [24,25]. Exosome biogenesis begins with invagination of the endosomal-limiting membrane and the formation of intraluminal vesicles (ILVs). ILVs move toward the inside of the cell to load cargo, which can include DNA (mitochondrial, single-stranded, and double-stranded), RNA (messenger RNAs, miRNAs, and non-coding RNAs), or lipids, resulting in a specialized cell compartment, known as a multivesicular body (MVB). Extracellular release of ILVs is coordinated by the fusion of MVBs with the plasma membrane, resulting in the release of ILVs as exosomes [3,26] (Figure 1). Given their endosomal origin, exosomes are enriched in protein families involved in the construction of ILVs; for example, tetraspanins, tumor susceptibility gene 101(Tsg101), and ALG-2 interacting protein-X (Alix). Furthermore, exosomes carry non-specific proteins, such as membrane combination and transfer proteins (e.g., annexins, Rab, and flotillins), major histocompatibility complex (MHC) proteins (e.g., MHC I and MHC II), heat shock proteins (e.g., Hsp70 and Hsp90), and cytoskeleton proteins (e.g., myosin, actin and tubulin) [27].

The endosomal sorting complex (ESCRT) comprises four fundamental complexes, ESCRT-0, I, II, and III, and is the apparatus with foremost responsibility for conveying particular cargos into ILVs of MVBs, facilitating protein reuse [28]. In addition to the ESCRT-dependent pathway, an ESCRT-independent route is also an available mechanism. For example, a few proteins, such as the Ras-related protein (Rab) family (Rab27a and Rab27b), four transmembrane-domain proteins (CD9, CD63, and CD81), and sphingomyelinase within the brain, also appear to be involved in lipid bi-layer formation, endosomal vesicle traffic, and vesicle release [29].

## 3. Functions of sEVs in Cancer

**Figure 2:**
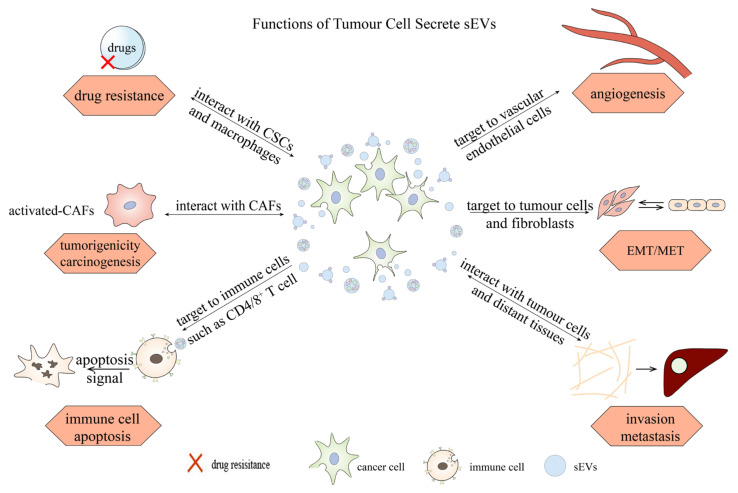
Six aspects of the roles sEVs in carcinogenesis. EMT/MET, epithelial-to-mesenchymal/mesenchymal-to-epithelial transition; sEVs, small extracellular vesicles; CAFs, cancer-associated fibroblasts; CSCs, cancer stem cells.

sEVs are among the most abundant carriers in nearly all body fluids, transporting different cargoes from an initial cell population to another, nearby or distant, and participating in various cell activities [3]. An increasing numbers of reports indicate that carcinoma-cell-derived sEVs transfer specific cargoes into recipient cells and modulate cancer processes; for example, angiogenesis, cell transformation, invasion, immune escape, and even drug resistance [30]. The specific roles of sEVs depend on their contents, including miRNAs, DNAs, tumor-derived proteins, and factors that modulate specific pathways [31]. The roles of sEVs in carcinogenesis can be categorized into six aspects, the role of sEVs in angiogenesis, epithelial-to-mesenchymal transition (EMT), invasion and metastasis, immune escape, cancer-associated fibroblasts (CAF), and drug resistance. As shown in Figure 2, sEVs derived from different cell populations can regulate different carcinogenic pathways via their distinct components [32]. We also explore the crucial roles of sEVs in drug resistance to probe the possibility for clinical application of sEVs against cancer recurrence and drug resistance [33].

### 3.1. The Role of sEVs in Angiogenesis

Angiogenesis, characterized as the arrangement of new blood vessels from a pre-existing vascular system, occurs during tissue development and growth, as well as in reaction to harm, to reestablish the blood supply of a tissue and facilitate wound healing [34]. sEVs provide recipient cells with pro- and antiangiogenic factors, to remodel them for roles in angiogenesis and carcinogenesis [35]. The contents of sEVs differ, as various cancers have unique angiogenic mechanisms. For example, in glioblastoma, several proangiogenic molecules are found in TEVs, including angiogenin, fibroblast growth factor, vascular endothelia growth factor, interleukin-6 (IL-6), among others. These molecules promote angiogenesis and enhance the malignancy of spongioblastoma [36]. Another proangiogenic molecule, annexin II, derived from breast cancer cells, can promote tumor neo-angiogenesis [37]. sEVs in body fluid from patients with pancreatic adenocarcinoma and colon cancer are loaded with considerable amounts of tetraspanin 8, which facilitates angiogenesis and metastasis [38,39]. Overall, angiogenesis relies on various proangiogenic or antiangiogenic molecules in sEVs, and sEVs are required for their transport from cancer cells to endothelial cells, and finally regulate the TME to enable tumor survival. 

### 3.2. The Role of sEVs in EMT

The more aggressive malignant tumor cells become, the more likely they are to migrate to distant sites. To help facilitate this, a new metastatic niche is needed at the distant site, and tumor epithelial cells must go through the process of EMT [33]. In tumors, the local influence of sEV-mediated cell signaling can promote cancer cell aggression. These features include morphological changes, which are related to migratory functions (EMT, cytoskeletal reorganization, and aggressive pseudopodia formation), motility, and basement membrane remodeling activities. Serum sEVs containing miR-200 can affect the infiltration and colonization potential of cancer cells, indicating that TEVs are associated with an aggressive phenotype in cholangiocarcinoma [40]. It is conceivable that, epithelioid cancer cells and mesenchymal cancer cells can be identified based on the different substances carried by their sEVs, which could affect cancer metastasis or resistance to treatment [41]. Although TEVs can reshape distant, metastasis-prone organs (promoting metastasis [41,42]), the exact and comprehensive roles of sEVs in metastatic sites are not fully understood [43].

### 3.3. The Role of sEVs in Invasion and Metastasis

sEVs play an indispensable role in cancer development, invasion, and metastasis [44]. For example, in gastric cancer (GC), sEVs promote liver-specific metastasis by carrying and transferring epidermal growth factor receptors [45]. sEVs can also contribute to tumor metastasis by improving the tumor-cell-establishing premetastatic niche and remodeling the extracellular matrix. The premetastatic niche is a prerequisite for tumor metastasis, in which TEVs can advance tumor angiogenesis and thrombosis by stimulation of endothelial cells [46]. TEVs can also convert mesenchymal stem cells into myofibroblasts, to enhance tumor metastasis [47]; moreover, sEVs can induce both cell transformation and activation of specific signaling molecules during the process of metastasis, such as the proto-oncogene tyrosine-protein kinase, Src, focal adhesion kinase (FAK), and neurotrophic tyrosine kinase receptor type 1 (TrkA), in recipient cells [48,49]. 

### 3.4. The Role of sEVs in Immune Escape 

TEVscan act as negative regulators of two molecules that function as T cell receptors, interleukin 2 receptor (IL-2R) and the T cell receptor, though inhibiting the expression and phosphorylation of Janus kinase (JAK) [50]. An intact JAK pathway is essential for the activity of cytokines, such as IL-2, IL-7, and IL-15, which share the gamma chain of IL-2R. Therefore, TEVs are detrimental to T cell proliferation and induce immune suppression by promoting regulatory T cell expansion and the demise of antitumor CD8^+^ effector T cells, thus contributing to tumor escape [50]. TEV-mediated signals lead to apoptosis of activated CD8^+^ T cells, which is related to cytochrome C discharge from mitochondria, early membrane changes in recipient cells, and DNA fragmentation [51]. TEVs target the PI3K/AKT pathway in activated CD8^+^ T cells. When CD8^+^ T cells are regulated by TEV, AKT dephosphorylation decreases, as do expression levels of BCL-2, BCL-xL, and MCL-1, accompanied by increased levels of the pro-apoptotic protein, BAX [52]. In summary, TEVs have biological activity in tumor immune suppression, which negatively influences the functions of various immune cells by types via one or more molecular pathways that cause change in target cells. 

### 3.5. sEVs and Cancer-Associated Fibroblasts (CAFs)

Cellular communications between cancer cells and surrounding stromal cells in the TME play important roles in regulating cancer progression and therapy responses [53]. Cancer-associated fibroblasts (CAFs) are crucial constituents of the TME that associate with cancer cells to advance tumorigenesis and movement [54], and sEVs can activate CAFs to influence the TME. In particular, prostate cancer cell-derived sEVs promote the activation of myofibroblasts [55]. TEVs also stimulate normal lung fibroblast activation (myofibroblast differentiation) in vitro [56] and advance the acquisition of myofibroblast-like characteristics in adipose tissue-derived mesenchymal stem cells [57]. These findings support that a role for TEVs in inducing CAF tumorigenicity. By contrast, CAF-derived sEVs can promote cancer progression by contributing to the chemoresistance of colorectal cancer (CRC) stem cells (CSCs) [58], and increasing the therapeutic resistance of breast cancer cells [59]. 

### 3.6. The Role of sEVs in Drug Resistance

With advances in pharmacology, anticarcinoma drugs have been rapidly developed and many patients now receive adequate treatment and have improved prognosis; however, drug resistance has emerged as a new problem. Like antibiological drugs, anticarcinoma drugs may have no effect in some cases, because of special mechanisms induced by tumor cells. Multidrug resistance proteins (MRPs) are key factors determining cancer drug resistance, which can also be affected by sEVs [60,61]. In one study, mesenchymal stem cell-derived sEVs were found to confer GC cell drug resistance both ex vivo and in vivo, mainly through their proteins, including multidrug resistance (MDR), MRP, and lung resistance-related protein [61]. In addition, the study showed that sEVs from doxorubicin-resistant osteosarcoma cells are likely taken up into auxiliary cells, where they invoke a doxorubicin-resistant phenotype, and multidrug-resistant osteosarcoma cells are able to spread their capacity resist the impact of doxorubicin treatment by exchanging sEVs carrying MDR-1 mRNA and P-glycoprotein [60].

#### 3.6.1. Transfer of Drug Resistance Mediated by Cancer Stem Cell sEVs

CSCs, a group of tumor cells in the TME with self-renewal and differentiation ability, are associated with drug resistance [62]. CSCs specifically express surface biomarkers, such as low levels of CD24 (CD24-/low) and high levels of CD44 (CD44^+^), allowing their selection using simple techniques [63,64]. In addition to the strong correlation between CSCs and tumor expansion, metastasis, and relapse, several molecular mechanisms are mediated by CSC-derived sEVs, such as activation of CAFs and the induction of EMT [65,66,67]. CSC-derived sEV miRNAs can contribute to the transfer of drug resistance to sensitive breast cancer cells. For example, miR-155 in CSC-derived sEVs may strengthen the resistance of breast cancer cells to paclitaxel and doxorubicin treatment [68]. Furthermore, the high level of miR-210 in pancreatic CSC sEVs from gemcitabine-resistant patients could transfer the resistant phenotype to gemcitabine-sensitive pancreatic cells [69]. 

#### 3.6.2. Macrophage Polarization and Drug Resistance

In addition to inducing CSC activity, drug resistance can also be realized by activation of tumor-associated macrophages (TAMs). TAMs belong to the most plentiful group of immune cells in the TME and are involved in immunosuppression, tumor angiogenesis, and cell resistance to chemotherapy [70,71]. TAM populations in the TME are related to poor prognosis in various cancers, with a larger population often correlated with worse prognosis [72]. Furthermore, the sEV-mediated transfer of TAM-derived miR-21 can confer resistance to cisplatin, and targeting sEV-mediated communication may be a promising new therapeutic strategy for patients with GC [73]. In colon cancer, miR-1246 with in carcinoma-derived sEVs induce macrophages to acquire a TAM phenotype [74]. Similarly, other studies have shown that TEVs can enhance macrophage transition to TAMs via miRNAs in several types of cancer, including ovarian [75,76], bladder [77], and lung [78] cancer. In conclusion, TAMs are associated with drug resistance.

## 4. sEVs as Biomarkers in Cancer

TEVs are both potential biomarkers for monitoring cancer progression and potential targets for future treatments. Given that TEVs are detectable in all body fluids, including blood, urine, and bronchial fluids [79], they can function as biomarkers in liquid biopsies. Here, we discuss the clinical application of sEVs-derived proteins and nucleic acids as biomarkers. Due to the limited techniques, lipids or other biomolecules in sEVs are not available for detection to effectively transform research results into clinical application [80]. Moreover, TEVs can also be used as non-invasive biomarkers to manage treatment responses. Here, we will highlight examples of the potential for use of sEVs as cancer biomarkers in clinical practice and provide a vision for future clinical diagnosis and prognosis of cancer.

### 4.1. sEVs Protein and Nucleic Acid Biomarkers

#### 4.1.1. Nucleic Acids in sEVs

miRNAs are RNA fragments of approximately 21–23 nucleotides that regulate gene expression in eukaryotic cells [81]. In cancer cells, miRNAs can also serve as biomarkers in cancer diagnosis. sEVs-derived miRNA can be detected for early cancer diagnosis, which may enhance both the sensitivity and specificity of tumor detection for lung cancer and pancreatic ductal adenocarcinoma (PDAC), for example [82,83]. In patients with hepatocellular carcinoma (HCC), researchers found that overexpression of sEV-derived miRNAs was associated with cancer diagnosis and prognosis [84]. The efficiency of different serum sEV-derived miRNAs for use in prognosis varies. For example, overexpression of serum exo-miR-215-5p is closely related to poor disease-free survival of patients with liver cancer, and can be used as a prognostic biomarker for HCC [85], however, the mechanisms underlying the activities of many miRNA remain unknown. Upregulation of circulating sEV-derived miRNA-373 [86], miRNA-1290, and miRNA-375 may have value for predicting prognosis of patients with prostate cancer [87].

Long-chain non-coding RNAs (lncRNAs) are a family of non-protein-coding RNAs (200 nt to 10 kb) with distinct expression in various diseases, including malignant tumors [88]. LncRNAs have vital roles in regulating gene expression, alternative splicing mechanisms, protein localization and activity, as well as cell substructure and protein complex formation through various interactions with DNA, RNA, and proteins [89,90]. Several independent studies have demonstrated that sEV-derived lncRNAs are involved in the proliferation of various cancers [91], as well as chemoresistance [92] and stemness [93] qualities. It is reported that lncRNA actin filament associated protein 1 antisense RNA 1(AFAP1-AS1) confers trastuzumab resistance of breast cancer cells via packaging into sEVs. Mechanistically, AFAP1-AS1 promotes AUF1-mediated activation of ERBB2 translation, causing increased HER-2 expression and trastuzumab resistance [94]. Additionally, the lncRNA UFC1 (E2-like ubiquitin-fold modifier conjugating enzyme 1) expression level was increased in the serum sEVs of patients with non-small cell lung cancer (NSCLC). High levels of UFC1 were associated with tumor infiltration. Importantly, another study found that sEV-transmitted UFC1 could bind to EZH2 to downregulate PTEN gene expression and activate PI3K/Akt signaling, thereby promoting NSCLC tumorigenesis [95]. 

#### 4.1.2. Proteins in Cancer sEVs

sEV proteins reflect their cellular origin and may help in cancer diagnosis. The intercellular transfer of oncoproteins by sEVs contributes to facilitating tumorigenesis [96,97]. sEV proteins are also useful in the dynamic monitoring of cancers. For example, compared with healthy control subjects or with PDAC who did not deteriorate after diagnosis and treatment, macrophage migration inhibitory factor is elevated in the circulating sEVs of patients with PDAC whose condition deteriorates [98]. Glypican-1(GPC-1) is a potential biomarker for pancreatic cancer diagnosis [99], and human leucine rich alpha-2-glycoprotein 1 (LRG1) in urinary sEVs is a potential biomarker for diagnosis of NSCLC [100]. 

### 4.2. Potential of sEVs as Cancer Biomarkers

#### 4.2.1. Lung Cancer 

Lung cancer is one of the most dangerous cancers and efficient methods for its diagnosis are lacking. Liquid biopsy of sEVs is a method with high accuracy and specificity for early diagnosis of lung cancer [101], and sEV-derived miR-96 is a potential serum biomarker for malignant lung cancer [102]. In addition, the LIM-domain-only protein 7 (LMO7) gene is a target of miR-96. Targeting the miR-96-LMO7 axis could be used to develop new diagnostic or therapeutic strategies [103]. Studies also have been conducted to identify biomarkers from TEVs that can distinguish between adenocarcinoma (AC) and squamous cell carcinoma (SCC). The results showed that TEV miRNAs include the AC-specific molecules, miR-181-5p, miR-30a-3p, miR-30e-3p, and miR-361-5p; as well as the SCC-specific miR-10b-5p, miR-15b-5p, and miR-320b, which can be isolated from the plasma of patients with lung cancer. Expression levels of these sEVs miRNAs are reduced and can distinguish AC from SCC [104]. Further, sEV-mediated transmission of UFC1 expression was upregulated in tumor serum sEVs from patients with NSCLC and high level of UFC1 were associated with tumor infiltration [95], while LRG1 is expressed at higher levels in urinary sEVs from patients with NSCLC [100].

#### 4.2.2. Breast Cancer

Breast cancer is a complex disease which is the second most common cause of cancer-associated death among women [105]. Therefore, it is fundamental to discover specific or sensitive biomarkers for early diagnosis and real-time monitoring of breast cancer. In one study, levels of miR-1246 and miR-21 were elevated in plasma sEVs from 921 patients with breast cancer compared with healthy patients [106], indicating that miR-1246 and miR-21 levels could potentially serve as a diagnostic biomarker for breast cancer. Furthermore, sEV-derived miR-1246 may inhibit Cyclin-G2 expression, thereby contributing to breast cancer progression. Thus, high levels of sEV-derived miR-1246 could be a prognostic biomarker for metastatic breast cancer [107]. One study revealed that the levels of sEV-derived endothelial locus-1 (Del-1) were significantly up-regulated in patients with breast cancer relative to healthy people, and could be used as new diagnostic biomarker for patients with breast cancer [108]. 

#### 4.2.3. Prostate Cancer

Prostate cancer is a malignant-epithelial tumor that usually appears in men over 50 years of age [109]. Currently, prostate cancer is most commonly clinically diagnosed by detecting the prostate-specific antigen (PSA); however, this method may miss some aggressive prostate cancers, and the diagnosis of prostate cancer requires new biomarkers [110]. One miRNA, miR-182, of the miR-183 cluster family, was detected in prostate cancer cell-derived sEV from serum [111], and miR-375 and miR-1290 have potential to predict the prognosis of castration-resistant prostate cancer [87]. A study investigating serum sEV miRNAs from patients with prostate cancer (*n* = 44) found that miR-1246 has potential to serve as a diagnostic marker, with a specificity of 100% and sensitivity of 75%, for differentiating healthy individuals from those with prostate cancer [112].

#### 4.2.4. Colorectal Cancer (CRC)

CRC is one of the most common malignant diseases globally and has a high mortality rate [113]. Consequently, screening and early detection of CRC are essential, and there is an urgent need to identify specific biomarkers for this cancer. CRC-cell-derived sEVs containing the lncRNA RPPH1 is up-regulated in plasma from patients with CRC and down-regulated after tumor resection [114]. Additionally, the expression of sEV-derived colorectal neoplasia differentially expressed-h (CRNDE-h) was increased in 148 patients with CRC [115]. In one sample set, seven miRNAs (let-7a, miR-1229, miR-1246, miR-150, miR-21, miR-223, and miR-23a) were significantly overexpressed in serum sEVs from patients with CRC [116]. Tetraspanin 1 (TSPAN1) is a cancer-related protein with a role in cell mitosis and leads to abnormal cell differentiation; TSPAN1 expression levels are significantly higher in patients with CRC than in healthy controls [117]. Hence, there is evidence that sEVs may be useful as effective diagnostic or prognostic tools in CRC.

#### 4.2.5. Gastric Cancer (GC)

GC is the fourth most common malignant tumor globally and the most common cause of cancer-related death [118]. A recent study demonstrated that sEVs miRNAs are effective biomarkers in GC, where the up-regulation of miR-374a-5p in serum from patients with GC is an indicator of poor prognosis [119]. Furthermore, miR-217 overexpression enhances GC cell proliferation and reduces sEV CDH1 levels; hence, the imbalance of miR-217 in plasma sEVs can be used as a biomarker for GC diagnosis and classification [120]. Similarly, the sEV-derived lncRNA, undifferentiated-type early GC (UEGC), is remarkably up-regulated in patients with early GC, and can assist in early diagnosis of GC [121]. 

#### 4.2.6. Liver Cancer

Liver cancer is classified into primary and metastatic types. HCC, the most common form of liver cancer, is a malignant tumor with high mortality rates [122]. Since liver cancer generally has no particular signs in its early stage, the optimal treatment period is often missed [123], as early diagnosis is imperative for effective HCC treatment. Clinical studies have demonstrated that serum sEV miR-9-3p levels in patients with HCC are significantly higher than those in healthy people; miR-9-3p can induce HCC cells by down-regulating the expression of fibroblast growth factor 5 (FGF-5), inhibiting ERK1/2-mediated proliferation, and repressing apoptosis [124]. Serum levels of miR-21 were higher in patients with HCC than those with chronic hepatitis B and healthy volunteers; however, the sensitivity of detection was much lower than that of sEV-derived miR-21. In addition, elevated serum sEV miR-21 levels were positively correlated with tumor stage in patients with HCC [125]. Furthermore, levels of the lncRNA HEIH were significantly higher in serum sEVs from patients with HCC than in those with chronic hepatitis C and liver cirrhosis [126]. Moreover, levels of the lncRNA, activated by tumor growth factor-β (lncRNA ATB), in serum sEVs from patients with HCC are also positively correlated with tumor TNM stage and volume [126]. Hence, there is potential for use of sEV components as tools for diagnosis or prognosis of liver cancer.

#### 4.2.7. Cervical Cancer

Cervical cancer remains a leading cause of cancer-related deaths among women in developing countries [127]. Accurate and effective early screening methods are conducive to the prevention and early detection of cervical cancer, thereby improving patient survival rates [128]. In one study, 121 plasma samples from patients with cervical cancer and patients with precancerous signs were subjected to sEV miRNA sequencing, which demonstrated reduced expression of let-7d-3p and miR-30d-5p in patients with cervical cancer. Those sEV miRNAs may be useful for early detection of cervical cancer [129]. In addition, researchers collected cervicovaginal lavage specimens from patients with cervical cancer and found that expression levels of the lncRNAs, HOTAIR, MALAT1, and MEG3 differed significantly in cervical-TEVs relative to controls, and thus, may be useful for early detection and diagnosis [130]. 

#### 4.2.8. Bladder Cancer (BC)

BC is the sixth most common malignant tumor in men and the seventeenth in women. Compared with healthy subjects, patients with BC have higher levels of sEVs in urine and serum samples. At different stages of disease, the sensitivity of TEVs in urine is higher than that of serum; however, serum is more specific and urine is more sensitive [131], which may be due to the high stability over time of sEVs, especially in the blood [132]. In one study, miR-21-5p, miR-141-3p, and miR-205-5p were detected as potential specific, non-invasive diagnostic tools for BC [133]. Similarly, the urine sEVs miRNAs, miR-19b1-5p, 136-3p, and 139-5p, are potential candidates for use in BC diagnosis [134]. 

#### 4.2.9. Diffuse Large B Cell Lymphoma

Diffuse large B cell lymphoma (DLBCL) is an aggressive malignant lymphoma [135] and the standard prognostic assessment tool, the international prognostic index (IPI), can only anticipate survival time, not the impact of treatment [135]. However, analysis of the miRNA profiles of specific sEVs derived from DLBCL cells show that levels of sEV-derived miR-99a-5p and miR-125b-5p were significantly increased in the serum of patients with DLBCL [136]. In addition, circulating sEV-derived miR-451a is down-regulated in DLBCL compared with healthy controls (*P*<0.000 1) [137], while expression of miR-210, miR-155, and miR-21 is high in DLBCL serum [138]. These sEVs miRNAs may be new targets for diagnosis or treatment.

#### 4.2.10. Pancreatic Cancer

Pancreatic cancer is the fourth leading cause of cancer deaths in the United States [1]. The high mortality rate is mainly due to late diagnosis and the aggressiveness of pancreatic cancer. At present, treatment options are limited [139,140]. Therefore, finding new strategies for early detection is key to improve the prognosis of patients with pancreatic cancer, especially those with localized disease. Glypican-1+ circulating sEVs (GPC1+crExos) showed 100% sensitivity and specificity for each stage of pancreatic cancer (carcinoma in situ, stage I, and stage II–IV), and has the potential for use as a noninvasive diagnosis and screening tool to detect the early stages of pancreatic cancer and promote curative surgical treatment [141]. Furthermore, miR-196a and miR-1246 are highly selectively enriched in sEVs derived from pancreatic cancer cells, and can be used as biomarkers for different local pancreatic cancer subtypes [142].

#### 4.2.11. Endometrial Cancer

Endometrial cancer (EC) is the sixth most common cancer among women worldwide [143], and most ECs are diagnosed early due to symptoms of postmenopausal uterine bleeding [144]. In one study that evaluated miRNA content in urine-derived sEVs miR-200c from isolated sEVs showed the largest fold-change value; hence, sEV-derived miR-200c may be useful as a preliminary diagnostic marker for EC [145]. In a meta-analysis of patients with EC, serum miR-21 was identified as a novel biomarker for EC, where higher serum miR-21 levels were detected in patients with benign lesions and EC than in healthy controls [146].

## 5. sEVs and Future Perspectives in the Clinic

**Figure 3:**
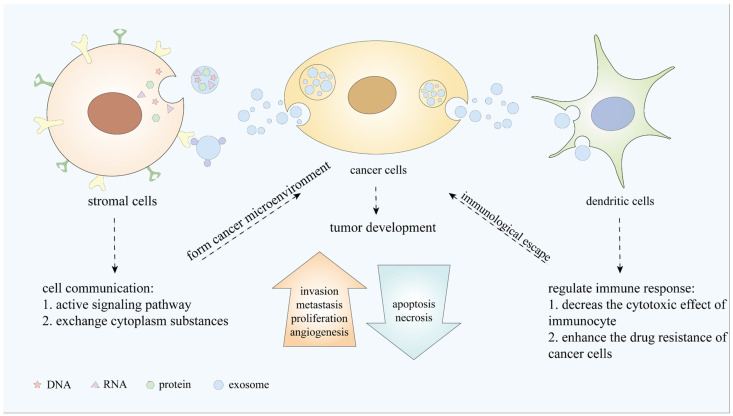
sEVs play an important role in cell communication, tumor development, and immune response.

As a subgroup of extracellular vesicles, sEVs play vital roles in intercellular communication and information transmission. Furthermore, sEVs from different origins regulate tumorigenesis via distinct mechanisms in almost all types of cancers. First, sEVs promote tumor angiogenesis and metastasis; sEV uptake up-regulates the expression of angiogenesis-related genes and enhances endothelial cell proliferation, migration, and growth [147]. Second, sEVs contribute to CAF transformation [148]. Third, sEVs can lead to the immune escape of tumor cells; the generation of an immunosuppressive environment is significant for cancer pathogenesis. Finally, sEVs protect cancer cells from the cytotoxic effects of chemotherapy drugs and transfer drug resistance to nearby cells [149]. TEVs are also necessary for regulation of immune cell physiological activity [150,151]. Circulating sEVs, derivedfrom the TME, contain proteins that promote immune tolerance, and thereby immune escape (Figure 3). 

Through the analysis of cancer patient body fluids, sEVs have been shown to have huge potential as noninvasive markers applicable to cancer diagnosis, screening, and monitoring, particularly using miRNAs and miRNA clusters (Table 3). sEV-derived miRNAs have the potential to be noninvasive biomarkers that indicate disease progression; for example, a group of sEVs miRNAs, including let-7a, miR-1229, miR-1246, miR-150, miR-21, miR-223, and miR-23a, can be used as diagnostic biomarkers for colon cancer [116]. In addition, among various sEVs, miRNA clusters are vital in cancer diagnosis. For example, the miR-17/92 cluster, which includes six miRNAs (miR-17-5p, miR-18a-5p, miR-19a-3p, miR-19b-1-5p, miR-20a-5p and miR-92a-1-5p) was selected as a potential diagnostic candidate marker for the diagnosis of non-NSCLC [152]. In many studies, sEVs containing specific miRNA clusters were genetically engineered for use in clinical practice, indicating the huge potential for the use of miRNA clusters in clinical cancer diagnosis and treatment [153].

As biomarkers, sEVs can provide rich, stable, sensitive, and specific biological information; they are a type of liquid biopsy specimen with high translational clinical value. Hence, the establishment of highly sensitive and rapid EV analysis technology is necessary for the development of liquid biopsies and methods for treatment with EVs. A highly sensitive and rapid analytic technology, termed ExoScreen, has been established to analyze surface proteins in extracellular vesicles from patient blood samples to identify CRC biomarkers. ExoScreen can monitor circulating EVs in serum without the need for purification steps. In addition to being a new liquid biopsy platform for detecting circulating EVs, ExoScreen can also help to diagnose various diseases and to identify biomarkers are important for new drug development [154]. 

## 6. Conclusions

With advances in precision medicine, traditional solid biopsy has shown considerable limitations, while the emergence of liquid biopsy has substantially compensated for these, providing a promising platform for non-invasive diagnosis and prognosis techniques. In this review, we described the biogenesis of sEVs and the main mechanisms of sEV-mediated transfer and chemical resistance. Understanding the molecular mechanisms involved in sEV biogenesis, and their roles in metastasis and chemoresistance, will provide insights into the design of new therapies for sEV-mediated tumor metastasis and chemoresistance. Nevertheless, sEVs are involved in numerous pathophysiological conditions. Currently, cancer treatment research based on sEVs still needs to solve the problem of sEV loading with therapeutic agents (including functional proteins, miRNAs and various chemotherapeutics). Furthermore, more clinical trials are needed to verify the feasibility of sEVs disease screening and monitoring biomarker.

## Figures and Tables

**Table 1:** The size of extracellular vesicles.

Vesicles	Size (nm)	Origin	Reference
Exosomes	30–100	endosomes	[5]
Microvesicles	100–1000	Plasma membrane	[8]
Apoptotic bodies	500–2000	Plasma membrane, endoplasmic reticulum	[9]

**Table 2:** The chemical characteristics of extracellular vesicles [5].

Characteristics	Recommended Nomenclature	
Physical characteristics	Size	Small: diameter <200 nm or <100 nm; Large and/or medium: >200 nm
	Density	Low; middle; high
Biochemical composition		e.g., CD63^+^/CD81^+^- EVs, Annexin A5-stained EVs, etc.
Conditions or cell of origin		e.g., podocyte EVs, hypoxic EVs, large oncosomes, apoptotic bodies, etc.

**Table 3:** The potential of sEVs as cancer biomarkers in clinical practice.

Cancers	sEVs Biomarkers	Analysis	Application	Sample Source	Reference
Bladder Cancer	miR-21-5p, miR-141-3p and miR-205-5p	qRT-PCR	diagnostic	urine	[133]
Bladder Cancer	miR-19b1-5p, 136-3p, 139-5p	qRT-PCR	diagnostic	urine	[134]
Breast cancer	miR-21	qRT-PCR	diagnostic	plasma	[106]
Breast cancer	miR-1246	qRT-PCR	diagnostic and prognosis	plasma	[107]
Breast cancer	miR-106a-5p, miR-106a-363 cluster (miR-19b-3p, miR-20b-5p and miR-92a-3p)	qRT-PCR	diagnostic	plasma	[155]
Breast cancer	miR-1280, miR-1260, and miR-720	qRT-PCR	diagnostic	serum	[156]
Breast cancer	Endothelial Locus-1 (Del-1)	ELISA	diagnostic	plasma	[108]
Cervical cancer	DEmiR (miR-30d-5p and let-7d-3p)	ddPCR	diagnostic	plasma	[129]
Cervical cancer	lncRNA HOTAIR, MALAT1	qRT-PCR	diagnostic and therapeutic	cervicovaginal lavage specimens	[130]
Cervical cancer	lncRNA MEG3	qRT-PCR	diagnostic and therapeutic	cervicovaginal lavage specimens	[130]
Colorectal cancer	lncRNA CRNDE-h	qRT-PCR	diagnostic	serum	[115]
Colorectal cancer	lncRNA RPPH1	qRT-PCR	diagnostic	plasma	[114]
Colorectal cancer	let-7a, miR-1229, miR-1246, miR-150, miR-21, miR-223and miR-23a	qRT-PCR	diagnostic	plasma	[116]
Colorectal cancer	TSPAN1	qRT-PCR	diagnostic	plasma	[117]
Diffuse large B cell lymphoma	miR-99a-5p and miR-125b-5p	qRT-PCR	prognosis	serum	[134]
Diffuse large B cell lymphoma	miR-451a	qRT-PCR	therapy	serum	[137]
Diffuse large B cell lymphoma	miR-155 and miR-21	qRT-PCR	diagnostic	serum	[138]
Endometrial cancer	miR-200c	qRT-PCR	diagnostic	urine	[145]
Endometrial cancer	miR-21	meta-analysis	prognosis	serum	[146]
Gastric cancer	miR-374a-5p;	qRT-PCR	prognosis	serum	[119]
Gastric cancer	miR-217	qRT-PCR	diagnostic	serum	[119]
Gastric cancer	lnc UEGC	qRT-PCR	diagnostic	plasma	[121]
Liver cancer	miR-21	qRT-PCR	diagnostic	serum	[125]
Liver cancer	lncRNA HEIH	qRT-PCR	diagnostic	serum	[126]
Liver cancer	lncRNA ATB	qRT-PCR	diagnostic and prognosis	serum	[126]
Lung cancer	miR-96	qRT-PCR	diagnostic or therapeutic	serum	[103]
Lung cancer (AC)	miR-181-5p, miR-30a-3p, miR-30e-3p and miR-361-5p	qRT-PCR	diagnostic	serum	[104]
Lung cancer (SCC)	miR-10b-5p, miR-15b-5p and miR-320b	qRT-PCR	diagnostic	serum	[104]
Lung cancer	lncRNA UFC1	qRT-PCR	prognostic	serum	[95]
Lung cancer	leucine-rich α-2-glycoprotein (LRG1)	proteomic identification	diagnostic	urinary	[100]
Pancreatic cancer	GPC1 + crExos	qRT-PCR	diagnostic	serum	[141]
Pancreatic cancer	miR-196a and miR-1246	qRT-PCR	prognosis	plasma	[142]
Prostate cancer	miR-82 of miR-183 cluster	qRT-PCR	prognostic	serum	[111]
Prostate cancer	miR-1290	qRT-PCR	prognostic	plasma	[87]
Prostate cancer	miR-1246	NanoString	diagnostic	serum	[112]

## Data Availability

Not applicable.

## Abbreviations

AC	adenocarcinoma
SCC	squamous cell carcinoma
LRG1	leucine rich alpha-2-glycoprotein 1
AFAP1-AS1	actin filament associated protein 1 antisense RNA 1
Del-1	Endothelial Locus-1
PCA3	prostate cancer antigen 3
RPPH1	ribonuclease P RNA component H1
CRNDE-h	colorectal neoplasia differentially expressed-h
TSPAN1	Tetraspanin 1
HOTTIP	HOXA transcript at the distal tip
lnc UEGC	lncRNA up-regulated in the sEVs of gastric cancer
HEIH	hepatocellular carcinoma up-regulated 51 EZH2-associated
ATB	activated by tumor growth factor-β
HOTAIR	HOX transcript antisense RNA
MALAT1	Metastasis associated in lung adenocarcinoma transcript 1
MEG3	Maternally expressed gene 3
LNMAT2	lymph node metastasis-associated transcript 2
GPC1 + crExos	glypican-1 + circulating exosomes
LGALS3BP	lectin galactoside-binding soluble 3 binding protein
